# Dysregulated autophagy-related genes in septic cardiomyopathy: Comprehensive bioinformatics analysis based on the human transcriptomes and experimental validation

**DOI:** 10.3389/fcvm.2022.923066

**Published:** 2022-08-02

**Authors:** Hua-Xi Zou, Bai-Quan Qiu, Ze-Yu Zhang, Tie Hu, Li Wan, Ji-Chun Liu, Huang Huang, Song-Qing Lai

**Affiliations:** ^1^Department of Cardiovascular Surgery, The First Affiliated Hospital of Nanchang University, Nanchang, China; ^2^Institute of Cardiovascular Diseases, Jiangxi Academy of Clinical Medical Sciences, The First Affiliated Hospital of Nanchang University, Nanchang, China; ^3^Department of Cardiovascular Surgery, Second Affiliated Hospital of Nanchang University, Nanchang, China; ^4^Institute of Nanchang University Trauma Medicine, The First Affiliated Hospital of Nanchang University, Nanchang, China

**Keywords:** sepsis cardiomyopathy, autophagy, transcriptome, key genes, database

## Abstract

Septic cardiomyopathy (SCM) is severe organ dysfunction caused by sepsis that is associated with poor prognosis, and its pathobiological mechanisms remain unclear. Autophagy is a biological process that has recently been focused on SCM, yet the current understanding of the role of dysregulated autophagy in the pathogenesis of SCM remains limited and uncertain. Exploring the molecular mechanisms of disease based on the transcriptomes of human pathological samples may bring the closest insights. In this study, we analyzed the differential expression of autophagy-related genes in SCM based on the transcriptomes of human septic hearts, and further explored their potential crosstalk and functional pathways. Key functional module and hub genes were identified by constructing a protein–protein interaction network. Eight key genes (CCL2, MYC, TP53, SOD2, HIF1A, CTNNB1, CAT, and ADIPOQ) that regulate autophagy in SCM were identified after validation in a lipopolysaccharide (LPS)-induced H9c2 cardiomyoblast injury model, as well as the autophagic characteristic features. Furthermore, we found that key genes were associated with abnormal immune infiltration in septic hearts and have the potential to serve as biomarkers. Finally, we predicted drugs that may play a protective role in SCM by regulating autophagy based on our results. Our study provides evidence and new insights into the role of autophagy in SCM based on human septic heart transcriptomes, which would be of great benefit to reveal the molecular pathological mechanisms and explore the diagnostic and therapeutic targets for SCM.

## Introduction

Sepsis is a life-threatening organ dysfunction caused by a deregulated host response to infection, which is one of the major and urgent public health challenges worldwide (1, 2). Despite recent advances in the treatment of sepsis, the morbidity and mortality of sepsis remain high in clinical practice (3, 4). Recent focus on the pathophysiology of sepsis has increasingly shifted from the primary infection foci to the abnormal host response (5). The abnormal host responses frequently activate or inhibit multiple downstream pathways rather than target the eradication of infection, which in turn leads to multiorgan dysfunction (6). Cardiac dysfunction is one of the common forms of sepsis-induced organ dysfunction, which is also known as septic cardiomyopathy (SCM) (7, 8). SCM is considered to be a major contributor to septic shock and is associated with increasing mortality (9, 10). Different from other types of cardiomyopathies, SCM has a unique pathophysiological situation, more insidious clinical features, and a lack of characteristic biomarkers, which make its precise management difficult in current clinical work (8, 11–13). Further exploration of the molecular biological mechanisms of SCM is urgently needed to advance the clinical management of sepsis (14).

Autophagy is a highly conserved biological process that provides cellular quality control to promote survival, allowing cells to be adaptive in response to physiological responses or mild stress, which has been demonstrated to be a master regulator of cardiac homeostasis and function (15–17). However, excessive or insufficient autophagy under severe pathological stress may lead to substantial self-degradation or accumulation of toxic materials, and eventually trigger cellular dysfunction and death (16, 18). The characteristics of dysregulated autophagy have been reported in various SCM-related studies (19, 20). In the cellular and animal models of experimental SCM, a significant increase in autophagosomes with bilayer membrane structures encasing subcellular organelles was observed by transmission electron microscopy (TEM), and excessive autophagic flow in cells was detected by transfection with mRFP-GFP-LC3 adeno-associated virus, as well as significant variations in the autophagy-specific markers LC3 and P62, supporting activated autophagy in experimental SCM models (21–24). Relatively, TRPC1 deletion could bring myocardial protection by attenuating LPS-induced excessive autophagy activation, as well as Astragaloside IV treatment (21, 22, 25). The double-edged role of autophagy has been reported in SCM, and this discrepancy is considered to be related to the different severities and stages of SCM (16). Nevertheless, the current understanding of the role of autophagy in the pathogenesis of SCM remains limited and uncertain (26, 27). There are differential observations in SCM-related animal and cellular experiments due to differences in experimental settings, sepsis severity, drug specificity, and timing of administration, which makes it more difficult to truly translate molecular biological findings into meaningful clinical strategies (27).

Exploring the molecular biology of disease based on the transcriptomes of human pathological tissues may bring the closest realistic insight, which has already been applied in various disease studies and transformed into clinical benefits (28–30). In this study, we analyzed the differential expression of autophagy-related genes (ARGs) in SCM based on the transcriptomes of human septic heart samples and further explored their potential crosstalk and functional pathways. Key functional module and hub genes were identified by constructing a protein–protein interaction (PPI) network, and the expression of hub genes was validated in a lipopolysaccharide (LPS)-induced H9c2 cardiomyoblast injury model, as well as the autophagic characteristic features. We subsequently explored the diagnostic capability and prognostic relevance of key genes for sepsis in whole blood transcriptomic data from independent cohorts. In addition, given the close association between SCM and the immune response, we performed immune correlation analysis on the identified key genes (8, 31). Finally, we predicted potential therapeutic drugs based on key genes for further exploration.

## Materials and methods

### Data collection

The microarray datasets GSE79962 and GSE54514 were downloaded from the NCBI-GEO database,^1^ and both datasets are based on human samples. The transcriptomic data of heart samples from 20 patients who died from sepsis and 11 from non-heart failure donors (non-failing hearts that matched the requirements of donor heart but declined for use in transplantation due to non-pathological factors) as controls were extracted from the GSE79962 dataset, which was performed on the GPL6244 platform. The transcriptomic data of whole blood samples (contain blood cells and plasma) from 35 septic patients (including 26 septic survivors and 9 septic non-survivors) admitted to the intensive care unit and 18 healthy controls within the first 24 h were extracted from the GSE54514 dataset, which was performed on the GPL6947 platform. After ID conversion, the median expression value was taken as the gene expression value when multiple probes corresponded to one gene. All expression data were log2 transformed and quantile normalized before further analyses.

### Identification of differentially expressed genes and differentially expressed ARGs

Differentially expressed genes (DEGs) in septic hearts were identified using the limma package (version 3.48.0) in R software (version 4.1), and the false discovery rate (FDR) was regulated by the Benjamini and Hochberg method. FDR < 0.05 and | log2FC| ≥ 0.5 were defined as the selection thresholds for DEG selection.

Autophagy-related genes were derived from three widely used autophagy-related databases – the Human Autophagy Database^2^ (32), the Autophagy Database^3^ (33), and the Human Autophagy Modulator Database^4^ (34). After deduplication of genes, the merged ARG set contained 1,167 ARGs, as listed in Supplementary Table 1. Differentially expressed ARGs (DEARGs) were extracted from the DEGs using the VennDiagram package (1.6.20) in R software.

### Gene set enrichment analysis

Gene set enrichment analysis (GSEA) using the GSEA software^5^ (35) was performed to observe the overall correlation between ARGs and septic heart. The autophagy-associated gene set contains 1,167 ARGs obtained as mentioned. Genes in the GSE79962 dataset were scored and ranked by expression value to calculate the enrichment score (ES). FDR < 0.05 was considered significant in GSEA.

### Gene ontology and kyoto encyclopedia of genes and genomes enrichment analyses

The identified DEARGs were subjected to Gene Ontology (GO) and Kyoto Encyclopedia of Genes and Genomes (KEGG) enrichment analyses using the clusterProfiler package (version 3.12.0) in R software. The results with FDR < 0.05 were considered significantly enriched by DEARGs.

### Protein–protein interaction network and identification of key modules and hub genes

As described in previous studies (28), the STRING database^6^ and Cytoscape software (version 3.8.2) were used to establish and visualize a PPI network of DEARGs. Functional key module was identified by the Cytoscape plugin MCODE (the parameters were set to default: degree cutoff = 2, node score cutoff = 0.2, K-core = 2 and max depth = 100). Another plugin, Cytohubba, was used to identify hub genes. The built-in MCC algorithm of Cytohubba assigned a value to each gene in the PPI network and ranked these genes by values. The top 10 genes were significant and regarded as hub genes.

### Cell culture and treatment

H9c2 cardiomyoblasts were purchased from the Cell Bank/Stem Cell Bank (Chinese Academy of Sciences, China) and cultured in high-glucose Dulbecco’s modified Eagle’s medium (H-DMEM) (HyClone, GE Healthcare Life Sciences, United States), supplemented with 10% fetal bovine serum (FBS) (Gibco, Thermo Fisher Scientific, United States). Cells were incubated at 37 °C under standard conditions (5% CO_2_, 95% humidity, 21% O_2_ concentration).

Before the treatment of cells, LPS (Sigma, China) was freshly dissolved in PBS, and 3-methyladenine (3-MA, MedChemExpress, China) was freshly dissolved in culture medium. As with previous studies (36, 37), the H9c2 cardiomyoblast injury model was induced by treatment with LPS (10 μg/ml) in the culture medium for 24 h, and additional 3-MA (5 μM) pretreatment for 24 h in the LPS + 3-MA group was used to inhibit autophagy, while the control group received solvent only. Every experiment was repeated at least three times independently.

### Cytotoxicity assay

Cytotoxicity was determined by the release of lactate dehydrogenase (LDH). Assays were performed according to the manual of the LDH kit (Beyotime, China). In brief, 120 μl of cell media was incubated with 60 μl of reaction mixture in a 96 well plate. After incubation for 30 min at room temperature, the absorbance was measured at 490 and 600 nm in a Spark^®^ multimode microplate reader (Tecan, Switzerland). The percent cytotoxicity was calculated by dividing the absorbance for the experimental wells by the absorbance for 100% cytotoxicity.

### Western blot analysis

The western blot protocol was modified from our previous work (38). Briefly, Whole-cell lysates were prepared in RIPA lysis buffer (Beyotime, China) containing 1% PMSF. After protein quantification using the BCA protein assay kit (Beyotime, China), equal amounts of total protein per sample were separated by 12% sodium dodecyl sulfate-polyacrylamide gel electrophoresis (SDS-PAGE) and transferred to PVDF membranes. Subsequently, the membrane was blocked with 5% non-fat dry milk at room temperature for 2 h, followed by overnight incubation at 4°C in a shaker with specific primary antibodies against LC3 (Proteintech #14600-1-AP, China, 1:1,000), P62 (Cell Signaling Technology #39749, United States, 1:1,000), and β-actin (Biosharp #BL005B, China, 1:2,000). The membrane was then incubated with secondary antibodies (Beyotime #A0208, China, 1:2,000) at room temperature for 2 h. The positive blots were detected using ultrahigh sensitivity ECL Kit (Beyotime, China) and imaged by FluorChem FC3 (ProteinSimple, United States). ImageJ software (NIH, United States) was used for densitometric scanning of the blots, and specific protein levels were calculated relative to that of β-actin.

### Immunofluorescence analysis

H9c2 cardiomyoblasts were seeded in six-well plates with glass coverslips. After incubation and treatment as described above, the cells were fixed in 4% paraformaldehyde for 10 min and blocked with 5% bovine serum albumin in PBS for 30 min at room temperature. Subsequently, the cells were incubated overnight at 4°C with a specific primary antibody against LC3 (Proteintech #14600-1-AP, China, 1:200), followed by incubation with a secondary antibody (Beyotime #A0423, China, 1:500) at room temperature for 1 h. Nuclei were stained with DAPI for 10 min. Finally, fluorescence images were captured using an Eclipse C1 fluorescence microscope (Nikon, Japan) at corresponding excitation wavelengths, and the images were processed with ImageJ software (NIH, United States).

### Transmission electron microscopy imaging

After incubation and treatment as described above, H9c2 cardiomyoblasts were scraped off, treated with 2.5% glutaraldehyde overnight at 4°C and then fixed with 1% osmium tetroxide for 2 h at room temperature. Subsequently, samples were further embedded and sectioned after dehydration with alcohol, then stained with uranyl acetate and lead citrate. Autophagosomes and autolysosomes were observed and imaged by TEM (Hitachi 7800, Japan).

### Quantitative real-time PCR

The Quantitative real-time PCR (qRT-PCR) protocol was as described in our previous study (28). Briefly, total RNA was extracted from the cells using TRIzol reagent (Invitrogen, United States), and the quality and yield of RNA were evaluated to confirm that the isolated RNA could be used to assess mRNA expression. Total RNA was reverse transcribed into cDNA using RevertAid MM (ThermoFisher Scientific, United States). Gene expression levels were quantified by Power SYBR Green PCR Master Mix (ThermoFisher Scientific, United States) using an Applied Biosystems StepOnePlus Real-Time PCR System (ThermoFisher Scientific, United States). The mRNA expression levels were normalized to ACTB and were calculated according to the 2^–ΔΔ*CT*^ method. All primers were designed and synthesized by Sangon Biotech Co. Ltd. (Shanghai, China). Detailed primer information can be found in Supplementary Table 2.

### Immune infiltration analysis

Immune infiltration analysis was performed using the Sangerbox platform^7^ to estimate the proportion of infiltrating immune cells in septic hearts and control hearts. Normalized gene expression data of the GSE79962 dataset were submitted to the immune infiltration calculation tool in Sangerbox, using the CIBERSORT algorithm to calculate the proportion of 22 immune cell species in each sample.

### Receiver operating characteristic curve analysis

Receiver operating characteristic (ROC) curves were used to assess the diagnostic capability of genes, which was quantified by calculating the area under the ROC curve (AUC). Genes with AUC > 0.6 were considered diagnostic, while genes with AUC > 0.8 were considered to have an excellent diagnostic capability.

### Potential therapeutic drug prediction

We used protein-drug interaction data from the DSigDB database^8^ (39) to predict potential autophagy-regulating drugs for SCM. FDR < 0.05 and combined score > 5,000 were used as the cutoff, and higher combined scores indicated stronger associations between drugs and target proteins. The chemical structures of the predicted drugs were identified through the Drugbank database^9^ and the PubChem database^10^ (40, 41).

### Statistical analysis

Statistical analyses were performed with GraphPad Prism (La Jolla, CA) and the Sangerbox platform. Comparisons between two groups were performed using *t*-test or signed-rank test depending on the different features of the data, while ordinary one-way ANOVA was used to analyze the differences between multiple groups. Correlation analysis was performed with Spearman’s correlation test. Data are presented as the mean ± SD. Benjamini–Hochberg FDR correction was used to correct *P* values, and FDR < 0.05 was considered to be statistically significant.

## Results

### Overall protocol of the study

**FIGURE 1:**
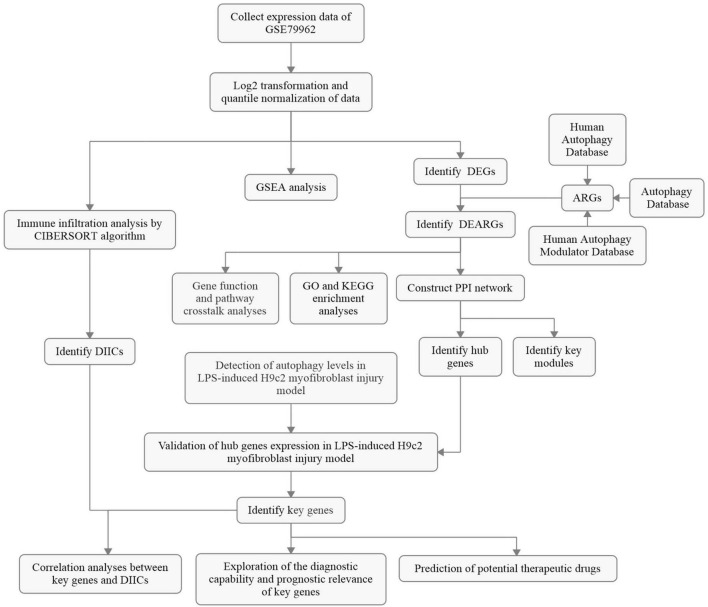
The overall protocol of this study. DEG, differentially expressed genes; ARG, autophagy-related gene; DEARG, differentially expressed ARG; GO, Gene Ontology; KEGG, Kyoto Encyclopedia of Genes and Genomes; PPI, protein–protein interaction; DIIC, differentially infiltrating immune cell; LPS, lipopolysaccharide.

The overall flowchart of the study is summarized in Figure 1. In total, transcriptomic data of 31 human heart samples (20 sepsis vs. 11 control) and 53 human whole blood samples (35 sepsis vs. 18 control) obtained from public databases were used in our study. All raw expression data were normalized as described above before further analyses, as shown in Supplementary Figure 1.

### Widespread dysregulation of autophagy-related genes is a feature of the altered transcriptome in the human septic heart

**FIGURE 2:**
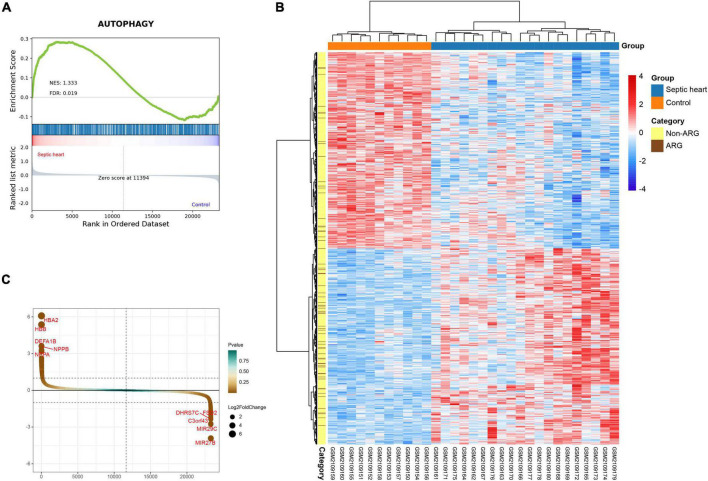
**(A)** Gene set enrichment analysis (GSEA) and identification of differentially expressed genes. GSEA on ARG set in GSE79962. **(B)** Clustered heatmap of DEGs in GSE79962 (FDR < 0.05 and |log2FC| ≥ 0.5). **(C)** Volcano plot of DEGs in GSE79962 (FDR < 0.05 and |log2FC| ≥ 0.5). ARG, autophagy-related gene; DEG, differentially expressed gene.

GSEA is a conventional approach to assess the overall relevance of a characteristic gene set to the disease transcriptome. To evaluate the overall variation of ARGs in the human SCM transcriptomes, we performed GSEA on the ARG set in GSE79962. The GSEA result, as shown in Figure 2A, indicates that the ARG set was significantly positive associated in septic heart transcriptomes compared with the control (NES = 1.333, FDR < 0.05), suggesting that widespread dysregulation of autophagy-associated genes is an essential feature of the altered transcriptome in human septic hearts, which provides human heart transcriptome based evidence for the correlation between autophagy and SCM.

### Identification of differentially expressed genes and differentially expressed ARGs

The clustering heatmap of DEGs shows significant differences in the transcriptomes between septic and control hearts (Figure 2B). A total of 980 DEGs were identified from GSE79962, of which 490 were upregulated in septic hearts, while 490 were downregulated (Figure 2C), as listed in Supplementary Table 3.

**FIGURE 3:**
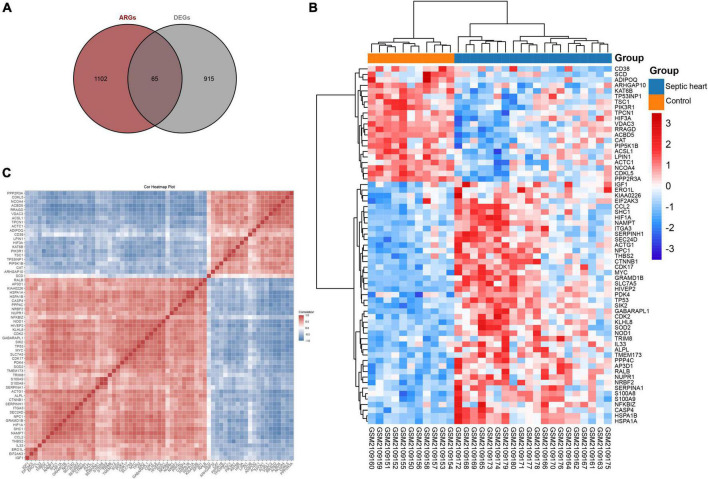
Identification of differentially expressed ferroptosis-related genes. **(A)** Venn diagram showing the overlap of genes between DEGs in GSE79962 and ARGs in autophagy-related databases. **(B)** Clustered heatmap of DEARGs in GSE79962. **(C)** Correlation heatmap of DEARGs in GSE79962, correlation coefficients are plotted with negative correlation (blue) and positive correlation (red). DEG, differentially expressed gene; DEARG, differentially expressed autophagy-related gene.

Since the GSEA results showed significant expression variations of ARGs in the septic heart transcriptomes, we further identified DEARGs. After deduplication of genes, a total of 1,167 ARGs were identified from autophagy-related databases. A total of 65 ARGs overlapped with the DEGs in GSE79962, which we identified as DEARGs for further analysis (Figure 3A), as listed in Supplementary Table 4. The clustered heatmap and correlation heatmap showed the expression differences of 65 DEARGs between septic and control hearts, as well as the correlation between DEARGs (Figures 3B,C).

### Gene ontology and kyoto encyclopedia of genes and genomes enrichment analyses

**FIGURE 4:**
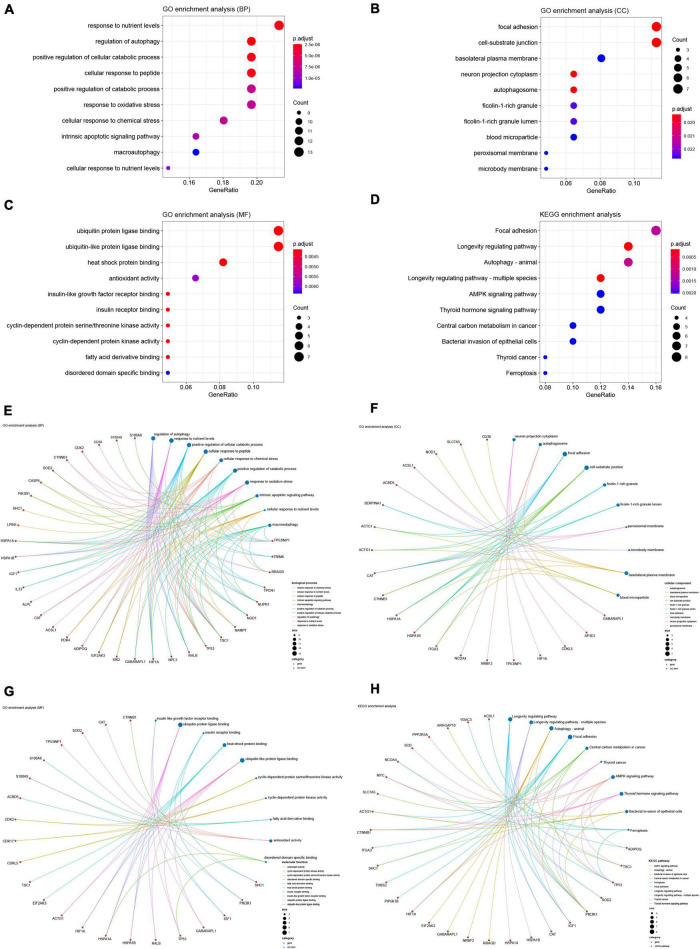
Gene Ontology (GO) and Kyoto Encyclopedia of Genes and Genomes (KEGG) enrichment analyses of differentially expressed ferroptosis-related genes. GO enrichment analysis of DEARGs in **(A)** the biological process category (BP); **(B)** the cellular component category (CC); **(C)** the molecular function category (MF). **(D)** KEGG enrichment analysis of DEARGs. Crosstalk analysis between DEFRGs and **(E)** gene functions in BP; **(F)** gene functions in CC; **(G)** gene functions in MF; **(H)** KEGG pathways. DEARG, differentially expressed autophagy-related gene.

We performed GO and KEGG enrichment analyses to explore the functions and related pathways of the DEARGs. Beyond the regulation of autophagy, DEARGs are also involved in the regulation of important biological processes (BP) such as cellular responses to oxidative and chemical stress, peptides, nutrient levels and apoptosis, as shown in Figure 4A. Given the close relationship between autophagy and some of these BPs in other biological circumstances (42–45), these BPs may be sequential to induce or be induced by autophagy in SCM. Cellular components (CC) localized by DEARGs include the autophagosome and some CCs affected by autophagy such as focal adhesion and cell-substrate junction (46, 47), as shown in Figure 4B. In the molecular function (MF), DEARGs mainly affect protein ubiquitination, an MF closely associated with autophagy (48, 49), as well as MFs such as antioxidant and protein kinase activity, heat shock protein and insulin receptor binding, which have also been reported to be associated with autophagy (50–53), as shown in Figure 4C. In the KEGG enrichment analysis, most of the DEARGs participated in autophagy, focal adhesion, AMPK signaling, ferroptosis, and longevity regulating pathways (Figure 4D), which echoed the results of the GO analyses. These pathways have been found to exist in crosstalk with autophagy regulation in many diseases, some of which have been reported to be involved in autophagy regulation in SCM experimental models (46, 54–56). We further analyzed the crosstalk between genes and different functions or pathways, and the results suggested that the regulatory role of ARGs in SCM may be the result of the crosstalk of multiple gene functions and pathways, as shown in Figures 4E–H.

### Construction of protein–protein interaction network and identification of key module and hub genes

**FIGURE 5:**
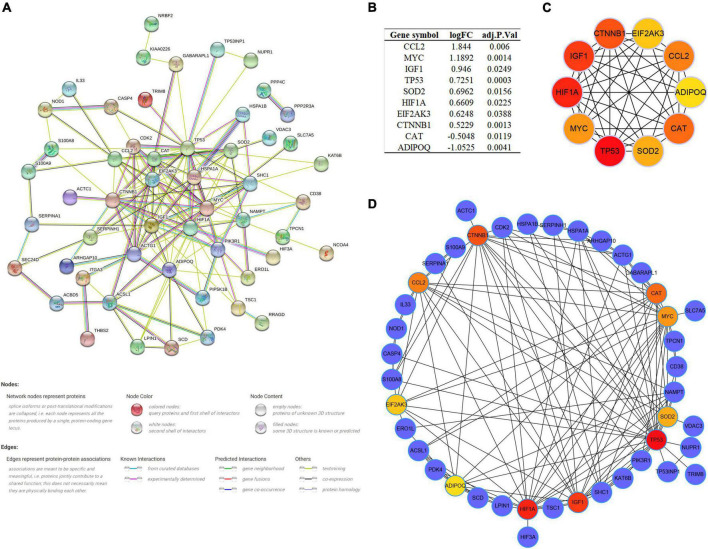
Protein–protein interaction (PPI) network and identification of hub genes. **(A)** PPI network of all DEARGs constructed by STRING database, no connected dots were hidden. **(B)** The differential expression of hub genes in septic hearts from GSE79962. **(C)** Crosstalk between top 10 hub genes ranked by MCC algorithm, the deeper color of the dot means that the rank order of the hub gene is more advanced. **(D)** Crosstalk between top 10 hub genes ranked by MCC algorithm and other DEARGs. DEARG, differentially expressed autophagy-related gene.

As multiple gene functions and pathways are involved and crosstalked in the regulatory role of ARGs in SCM, we constructed and analyzed the PPI network of DEARGs using the STRING database and Cytoscape software to find the functional module and key genes among them (Figure 5A). Finally, one key module was identified by the MCODE plugin (Supplementary Figure 2), and the top 10 genes (CCL2, MYC, IGF1, TP53, SOD2, HIF1A, EIF2AK3, CTNNB1, CAT, and ADIPOQ) ranked by the MCC algorithm of the Cytohubba plugin were selected as hub genes. Interestingly, the key module completely overlapped with the hub genes we identified, which further demonstrated that these hub genes are the major functional clusters of DEARGs. Figure 5B shows the differential expression of hub genes in septic hearts, while Figures 5C,D show the multiple associations between hub genes and with other DEARGs. The scores of all DEARGs in the PPI network calculated by the Cytohubba plugin are listed in Supplementary Table 5.

### Altered autophagy level and validation of hub gene expression in the LPS-induced H9c2 myofibroblast injury model

**FIGURE 6:**
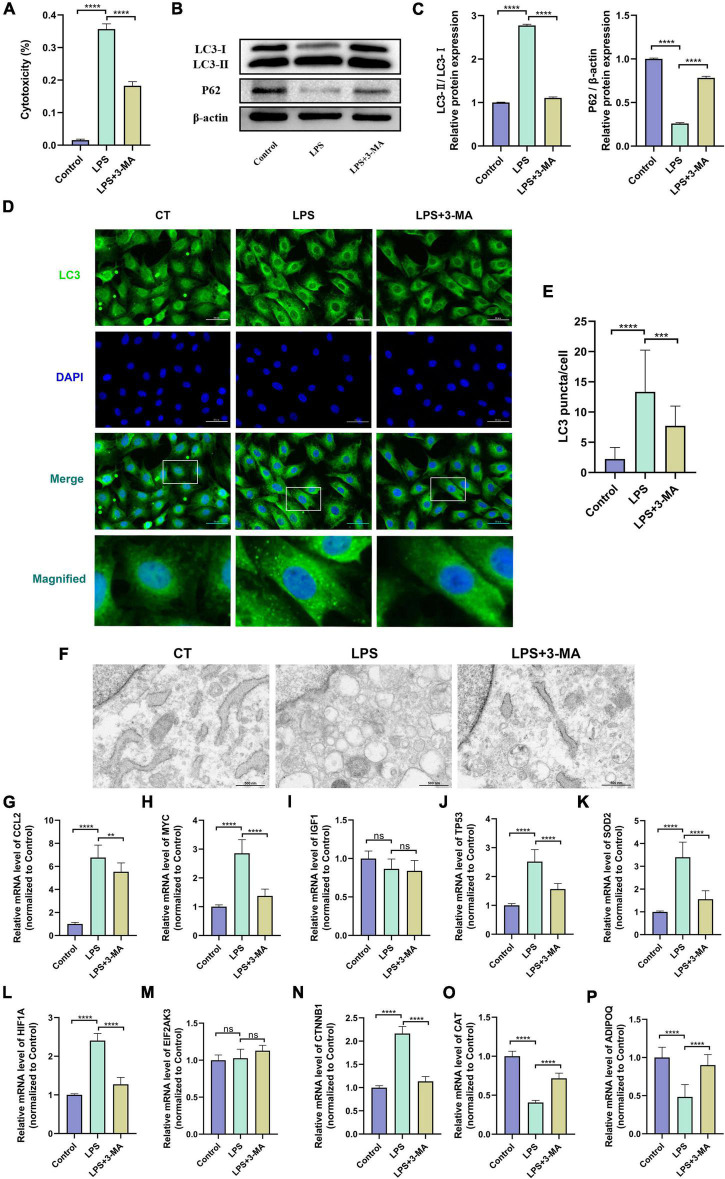
**(A)** Altered autophagy level and validation of hub gene expression in LPS-induced H9c2 myofibroblast injury model. H9c2 myofibroblasts were treated with LPS (10 μg/ml) for 24 h in the presence or absence of 3-MA (5 μM) pretreatment, followed by subsequent experiments. **(A)** Cytotoxicity determined by the release of lactate dehydrogenase. **(B)** Representative western blotting bands of LC3 and P62. **(C)** The LC3-II/I ratio and the relative protein levels of P62 estimated by ImageJ software. **(D)** Representative images of LC3B immunofluorescence in H9c2 myofibroblasts. **(E)** The Ratio of the number of LC3 puncta to the number of cells in each group. **(F)** Representative images of transmission electron microscopy in H9c2 myofibroblasts. **(G–P)** mRNA expression levels of hub genes detected by qRT-PCR. LPS, lipopolysaccharide; 3-MA, 3-methyladenine. ^**^p < 0.01, ^***^p < 0.001, ^****^p < 0.0001, ns = not significant. Data are presented as the mean ± SD, *n* ≥ 3.

The LPS-induced H9c2 myofibroblast injury model is a commonly used *in vitro* experimental model of SCM, which we used here to reflect the changes of autophagy level and hub gene expression in SCM (54, 57). The cytotoxicity assay showed increased cytotoxicity in the LPS-treated H9c2 myofibroblasts, which could be significantly alleviated by pretreatment with the autophagy inhibitor 3-MA (Figure 6A). Next, we examined the protein levels of LC3 and P62 in H9c2 myofibroblasts, which are widely used as indicators of autophagic flux. We found that LPS treatment resulted in an increase in the ratio of LC3-II/I as a marker of autophagy and a decrease in the expression of the autophagic substrate P62, which implies an increase in cellular autophagic flux (Figures 6B,C). Immunofluorescence results showed that LPS treatment increased LC3 positive puncta in the cytoplasm of H9c2 myofibroblasts, while TEM similarly observed an increase in autophagosomes and autolysosomes (Figures 6D–F). These results further demonstrated that LPS treatment induced increased autophagy levels in H9c2 myofibroblasts. Correspondingly, 3-MA pretreatment inhibited these autophagic features to some extent.

*In vitro* experiments and the transcriptomes of human samples consistently suggest the presence of dysregulated autophagy in SCM. We then verified the transcript levels of hub genes in the LPS-induced H9c2 myofibroblast injury model. qRT-PCR results showed that 8 of the 10 hub genes exhibited consistent trends in the transcriptomes of human samples after LPS treatment and could be significantly alleviated by 3-MA pretreatment, suggesting their involvement in regulating autophagy in SCM (Figures 6G–P). Thus, we identified these eight genes (CCL2, MYC, TP53, SOD2, HIF1A, CTNNB1, CAT, and ADIPOQ) as key genes regulating autophagy in SCM.

### Immune infiltration analyses

**FIGURE 7:**
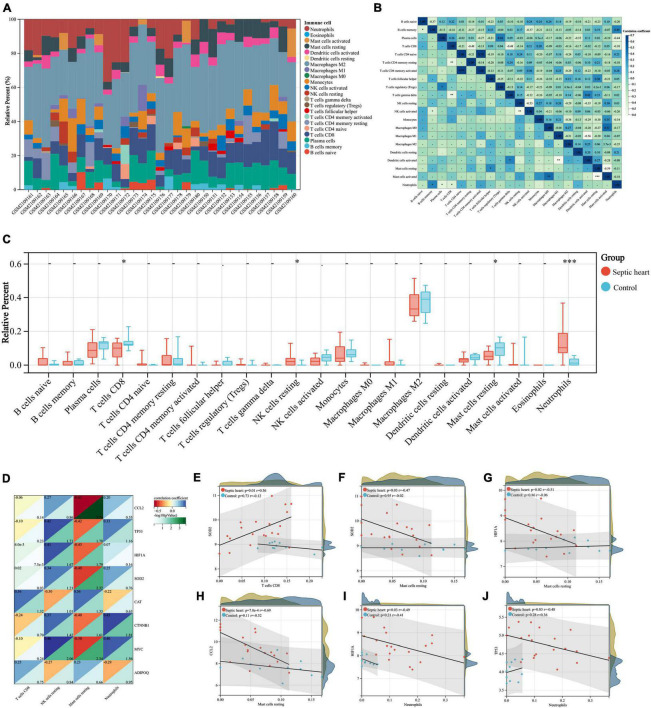
**(A)** Correlation analyses between key genes and DIICs. The proportion of infiltrating immune cells of the samples from GSE79962 by the CIBERSORT algorithm. **(B)** Correlation heatmap of DIICs in GSE79962, correlation coefficients are plotted with negative correlation (green) and positive correlation (blue). **(C)** Comparison of infiltrating immune cells between septic and normal hearts in GSE79962. **(D)** Correlation heatmap of DIICs and key genes in GSE79962, correlation coefficients are plotted with negative correlation (red) and positive correlation (blue), while *p* values are negatively correlated with greenness. **(E–J)** Linear regression plots presenting the significant correlation between key genes and DIICs. DIIC, differentially infiltrating immune cell. *p < 0.05, ^**^p < 0.01, ^***^p < 0.001, ns = not significant.

The important role of the immune response in the pathological mechanism of SCM has been demonstrated, therefore we performed immune infiltration analysis in an attempt to explore the crosstalk between ARGs and the immune response in SCM (8, 19, 58). The proportion of infiltrating immune cells in the heart samples from the GSE79962 dataset was estimated by the CIBERSORT algorithm (Supplementary Table 6) and then visualized (Figure 7A). The clustering heatmap shows the differential proportions of infiltrating immune cells between sepsis and control heart samples from the GSE79962 dataset (Supplementary Figure 3), while the correlation heatmap shows the correlation between different infiltrating immune cells (Figure 7B). By comparing the proportion of infiltrating immune cells in septic and control heart samples, we found a significant decrease in the proportion of CD8^+^ T cells and resting mast cells and a significant increase in the proportion of resting NK cells and neutrophils in septic hearts (Figure 7C), thus we identified them as differentially infiltrating immune cells (DIICs). We further observed the correlation between key genes and DIICs by linear regression analysis (Figure 7D) and found that in septic hearts, the proportion of CD8^+^ T cells was significantly positively correlated with the expression of SOD2; the proportion of resting mast cells was significantly negatively correlated with the expression of SOD2, HIF1A, and CCL2; and the proportion of neutrophils was significantly negatively correlated with the expression of HIF1A and TP53, while no significant correlation was present between the expression of key genes and the proportion of DIICs in control hearts (Figures 7E–J). These results suggest that the key genes we identified not only regulate cardiomyocyte autophagy but also participate in regulating the infiltration of immune cells in SCM.

### Exploration of the diagnostic capability and prognostic relevance of key genes

**FIGURE 8:**
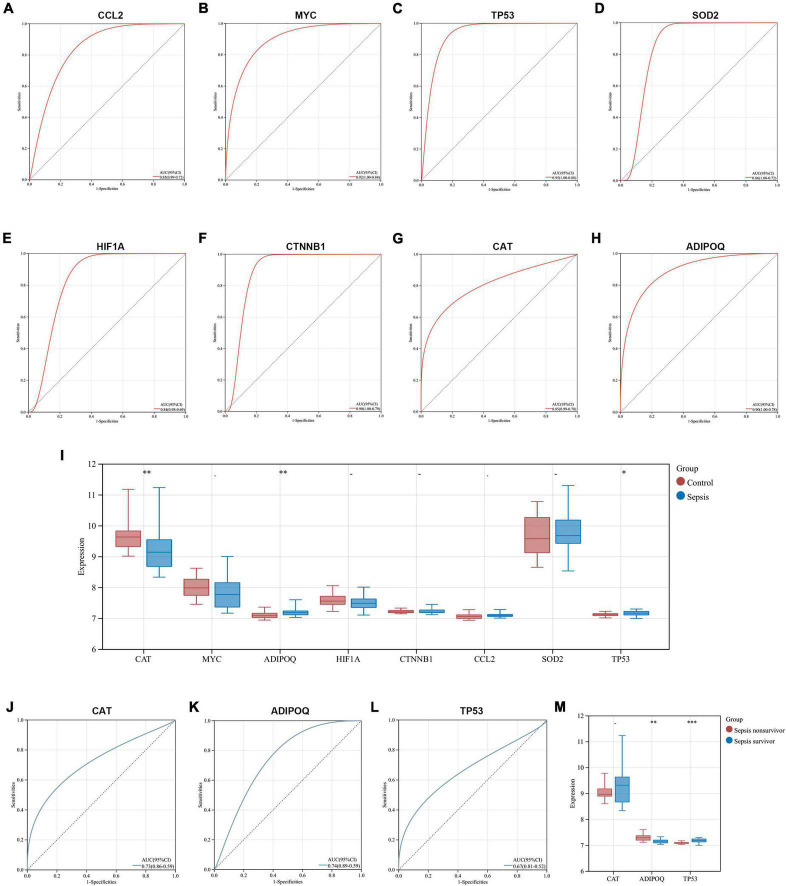
Exploration of the diagnostic capability and prognostic relevance of key genes. **(A–H)** ROC curves show the diagnostic capability of key genes for SCM in heart samples from GSE79962. **(I)** Comparison of key gene expression in whole blood samples from sepsis and healthy controls in GSE54514. **(J–L)** ROC curves show the diagnostic capability of CAT, ADIPOQ, and TP53 for sepsis in whole blood samples from GSE54514. **(M)** Comparison of CAT, ADIPOQ, and TP53 expression in whole blood samples from sepsis survivors and non-survivors in GSE54514. ROC, receiver operating characteristic; AUC, area under the ROC curve. *p < 0.05, ^**^p < 0.01, ^***^p < 0.001.

We explored the diagnostic capability of key genes in heart samples from the GSE79962 dataset and whole blood samples from the GSE54514 dataset, respectively. In the heart samples, the results of ROC analysis showed that all key genes exhibited excellent diagnostic capability (AUC > 0.8), which further identified the essential association between key genes and SCM (Figures 8A–H). Given that heart samples are difficult to obtain in clinical work, we then analyzed the expression and diagnostic capacity of key genes in whole blood samples. CAT and TP53 showed significant expression changes (downregulated and upregulated, respectively) in whole blood samples from sepsis patients, which was consistent with the trend in the heart samples, while ADIPOQ showed significantly opposite trends. The expression of other key genes did not differ significantly in whole blood samples from sepsis patients and healthy controls (Figure 8I). All three differentially expressed key genes exhibited diagnostic capability but seemed to be less than excellent (0.6 < AUC < 0.8), as shown in Figures 8J–L. In addition, we compared the expression of these three key genes in the subgroups of sepsis survivors and non-survivors, found that CAT showed a lower expression in the whole blood samples of sepsis non-survivors, but not significantly, while ADIPOD expression in sepsis non-survivors was significantly higher, and TP53 expression was significantly lower (Figure 8M).

These interesting results suggest that ARGs in SCM have the potential to be biomarkers of sepsis, however, since SCM is just one of the multiple organ dysfunctions caused by sepsis, its pathological molecular features may not well directly reflect the systemic state of septic patients. Further studies are needed to clarify and select biomarkers, especially those related to prognosis, considering that autophagy plays a double-edged role in the systemic state of sepsis.

### Potential autophagy-regulating drugs prediction

**FIGURE 9:**
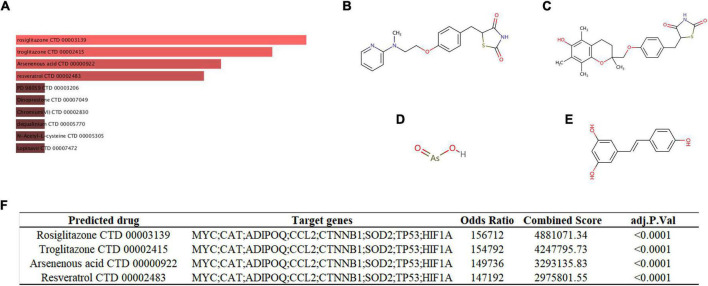
**(A)** Targeted drugs prediction. The top 10 predicted targeted drugs ranked according to combined score in the DSigDB database. The chemical structures of rosiglitazone **(B)**, troglitazone **(C)**, arsenous acid **(D)**, and resveratrol **(E)**. **(F)** Detailed prediction information of drugs with strong drug-target correlation.

We used the DSigDB database to predict potential autophagy-regulating drugs that are related to key genes, which may potentially treat SCM by modulating autophagy. Finally, 54 potential autophagy-regulating drugs were predicted, and the combined score and corresponding target genes are listed in Supplementary Table 7. Figure 9A shows the top 10 predicted drugs ranked according to combined score, especially the top four drugs – rosiglitazone (Figure 9B, combined score = 4,881,071), troglitazone (Figure 9C, combined score = 4,247,796), arsenous acid (Figure 9D, composite score = 3,293,136), and resveratrol (Figure 9E, composite score = 2,975,802), which had strong drug-target correlation (FDR < 0.001, composite score > 2 × 10^6^), as shown in Figure 9F.

## Discussion

As an essential organ dysfunction caused by sepsis, SCM is of great concern due to its uncertain clinical diagnosis and serious effect on the prognosis of sepsis, which urgently requires the identification of pathophysiological mechanisms to provide meaningful diagnostic and therapeutic targets for clinical work (58, 59). Dysregulated autophagy has been reported in SCM as a pathological mechanism of interest, however, its molecular biological mechanisms remain unclear (19). In the present study, we performed comprehensive bioinformatics analysis and experimental validation of dysregulated ARGs in the human septic heart transcriptomes were performed to explore the role of autophagy in SCM.

GSEA identified widespread dysregulation of ARGs in septic hearts, which corroborates with previous studies, suggesting that dysregulated autophagy may be an important pathological mechanism in SCM (60, 61). GO enrichment analysis further reveals the biological processes, cellular component localization, and molecular functions of ARGs in SCM. Most GO terms enriched by ARGs are strongly associated with autophagy. Autophagy can be activated in response to oxidative and chemical stress, nutrient deprivation, while dysregulated autophagy can induce oxidative stress (62, 63). Activation of autophagy can induce or antagonise apoptosis and thus have a completely different effect on cells (45, 64). Autophagy also plays an important role in the disassembly and turnover of focal adhesion (65, 66). Protein ubiquitination, antioxidant and protein kinase activity have been reported to be the main functions of many molecules participating in the regulation of autophagy, which are essential for autophagy activation and function (52, 67, 68). The focal adhesion and longevity regulating pathways crosstalk with autophagy regulation in many biological environments, yet have not been studied in SCM (69–71). We believe that this crosstalk between pathways deserves attention for understanding the molecular biological mechanisms affecting and being affected by dysregulated autophagy in SCM.

After constructing the PPI network, we identified the hub genes among the DEARGs, and eight of them were identified as key genes involved in the regulation of autophagy in SCM after validation in the *in vitro* experimental model of SCM. All key genes have reliable evidence supporting their involvement in the regulation of autophagy in different biological environments (32–34), of which HIF1A is involved in the positive regulation of autophagy (72); CCL2, SOD2, CTNNB1, CAT, and ADIPOQ in the negative regulation of autophagy (73–77); while MYC, and TP53 are involved in the bidirectional regulation of autophagy (78–81). Interestingly, despite the septic heart being in an overall overactivated state of autophagy, the expression of CCL2, SOD2, and CTNNB1, which are negative regulators of autophagy, was significantly increased. We consider that this may result in the following reasons – they may have an undiscovered bidirectional regulation on autophagy; they may be involved in biological processes related to autophagic processes in the SCM but not directly regulating autophagy; they may act as antagonists of protective autophagy in the SCM. Some of these key genes have been found to be important targets in the pathological development of SCM, while regulation of autophagy may be their undiscovered molecular biological function in the development of SCM, but further studies are required to clarify the specific regulatory mechanisms (82–85).

The key genes we identified are not autophagy-specific, they are also involved in many biological processes that are thought to be associated or unassociated with autophagy. While our study identified the association of key genes with autophagy in experimental models of SCM, their multiple roles in biological processes are noteworthy, which may be key to uncovering the interaction of autophagy with other biological processes in SCM. Some of the autophagy-specific genes were differentially expressed in the septic hearts we studied, e.g., ATG2A (FDR = 0.044), MAP1LC3B2 (FDR = 0.0018). Interestingly, partial autophagy-specific genes were not differentially expressed in septic hearts, we consider that the absence of significant alterations in the transcriptome of these genes may attributed to them being involved molecules in the autophagic terminal process and altered mainly at protein expression levels.

It is well known that immune and inflammatory responses play a critical role in the pathogenesis of SCM, and the inappropriate regulation of the immune system is inextricably linked to the pathological development of SCM (86, 87). Autophagy has been demonstrated to have an important effect on the homeostasis, function, differentiation, and survival of immune cells, the relevance of autophagy and immune infiltration in SCM is of concern but not yet clear (88, 89). In the septic heart samples we analyzed, the proportions of infiltrating CD8^+^ T cells and resting mast cells were reduced, whereas the proportions of infiltrating resting NK cells and neutrophils were increased, which was also partially observed in previous studies (90–92). We identified some significant correlations between the expression of key autophagy-related genes and the infiltrating proportion of immune cells in septic hearts, which was not present in control hearts. This may be the crosstalk between dysregulated autophagy and abnormal immune responses in SCM. Nonetheless, further functional clarification of the relationship between autophagy and immune infiltration in SCM is necessary, since some immune cells have both pro-inflammatory and anti-inflammatory biological functions, and the immune infiltration in sepsis varies among individuals and disease stages (93, 94).

Exploring effective therapeutics based on genes that play a key role in pathology has always been the focus of researchers (4, 14). Here, we predicted potential autophagy-regulating drugs for SCM based on the key genes we identified, in which rosiglitazone, troglitazone, arsenous acid and resveratrol presented high drug-target correlations. Arsenous acid has not been studied in sepsis, thus it cannot be determined whether it is beneficial or detrimental to SCM. Rosiglitazone and troglitazone, as thiazolidinedione class drugs, have been shown to provide protective effects in sepsis, including improved survival and alleviated organ dysfunction (95, 96). Moreover, rosiglitazone has been found to alleviate sepsis-related cardiac dysfunction and mortality by activating peroxisome proliferator-activated receptor-γ and inhibiting TNF-α expression (97, 98). Resveratrol, a natural phenolic compound, has also been found to have a significant protective effect in sepsis and protects the heart in sepsis by activating the Nrf2 and PI3K/AKT/mTOR pathways and inhibiting the NF-κB pathway (99–101). Autophagy is a potential regulatory mechanism for the role of these protective drugs in SCM, but it has not yet been explored.

Although our study provides evidence and new insights from dysregulated ARGs in human septic heart transcriptomes to explore the role of autophagy in SCM, there are some limitations. The role of autophagy in SCM is currently highly controversial; both impaired and excessive autophagy has been reported in numerous studies in various experimental models, as well as attempts to treat them in various ways. However, none of the studies have made direct observations on autophagic characteristics in the human SCM heart, which is also a limitation of our study. Given the controversial role of autophagy in SCM, direct observation of autophagy characteristics in human heart samples is urgently needed for investigating autophagy in SCM. Besides, the human septic heart transcriptomes we used were derived from patients who died from sepsis, which may only be representative of SCM patients with severe or poorly prognosis, and whether autophagy differs in the pathology of SCM across conditions and disease stages remains to be clarified, since we do notice that some studies have reported inhibition of autophagy in SCM (16). Further molecular functional investigations of the identified key genes regulating autophagy in SCM and observation of whether circulating and myocardial cytokines have synergistic or antagonistic effects on the regulation of autophagy in SCM are critical to find meaningful diagnostic and therapeutic targets. Therefore, further *in vivo* validation and functional research is required to translate our findings into clinical benefits.

## Conclusion

We found widespread dysregulation of ARGs in human septic hearts, and further bioinformatics analyses revealed that dysregulated ARGs were mainly localized in focal adhesion and cell-substrate junction, and affected response to nutrient levels, regulation of autophagy, and positive regulation of cellular catabolic process, possibly by regulating ubiquitin and ubiquitin-like protein ligase binding. Key genes (CCL2, MYC, TP53, SOD2, HIF1A, CTNNB1, CAT, and ADIPOQ) among dysregulated ARGs were identified and validated using *in vitro* experiments, and also found to be associated with abnormal immune infiltration in the septic heart and have the potential to serve as biomarkers. Furthermore, we predicted that rosiglitazone, troglitazone, and resveratrol may provide protection by modulating autophagy in SCM. Our study provides evidence and new insights into the role of autophagy in SCM based on human septic heart transcriptomes, which is of great benefit to revealing the pathobiological mechanisms and exploring the diagnostic and therapeutic targets for SCM.

## Data availability statement

The datasets presented in this study can be found in online repositories. The names of the repository/repositories and accession number(s) can be found in the article/Supplementary material.

## Ethics statement

GEO database belong to public databases, and the patients involved in the database have obtained ethical approval. Users can download relevant data for free for research and publish relevant articles. There are no other required ethical statements.

## Author contributions

S-QL and HH: conception and design. J-CL and LW: administrative support. S-QL and Z-YZ: collection and assembly of data. H-XZ and B-QQ: data analysis and interpretation. H-XZ and TH: cell experiments. All authors wrote the manuscript, final approval of manuscript, and have read and agreed to the published version of the manuscript.

## Footnotes

1
https://www.ncbi.nlm.nih.gov/geo/

2
http://www.autophagy.lu/index.html

3
http://www.tanpaku.org/autophagy/index.html

4
http://hamdb.scbdd.com

5
http://software.broadinstitute.org/gsea/index.jsp

6
https://string-db.org/

7
http://vip.sangerbox.com/

8
http://dsigdb.tanlab.org/

9
http://www.drugbank.ca

10
http://pubchem.ncbi.nlm.nih.gov
