# Supplementary figures and images for: Dysregulated autophagy-related genes in septic cardiomyopathy: Comprehensive bioinformatics analysis based on the human transcriptomes and experimental validation
Source: Front Cardiovasc Med. 2022 Aug 2;9:923066. doi: 10.3389/fcvm.2022.923066 (PMC9378994; doi:10.3389/fcvm.2022.923066)

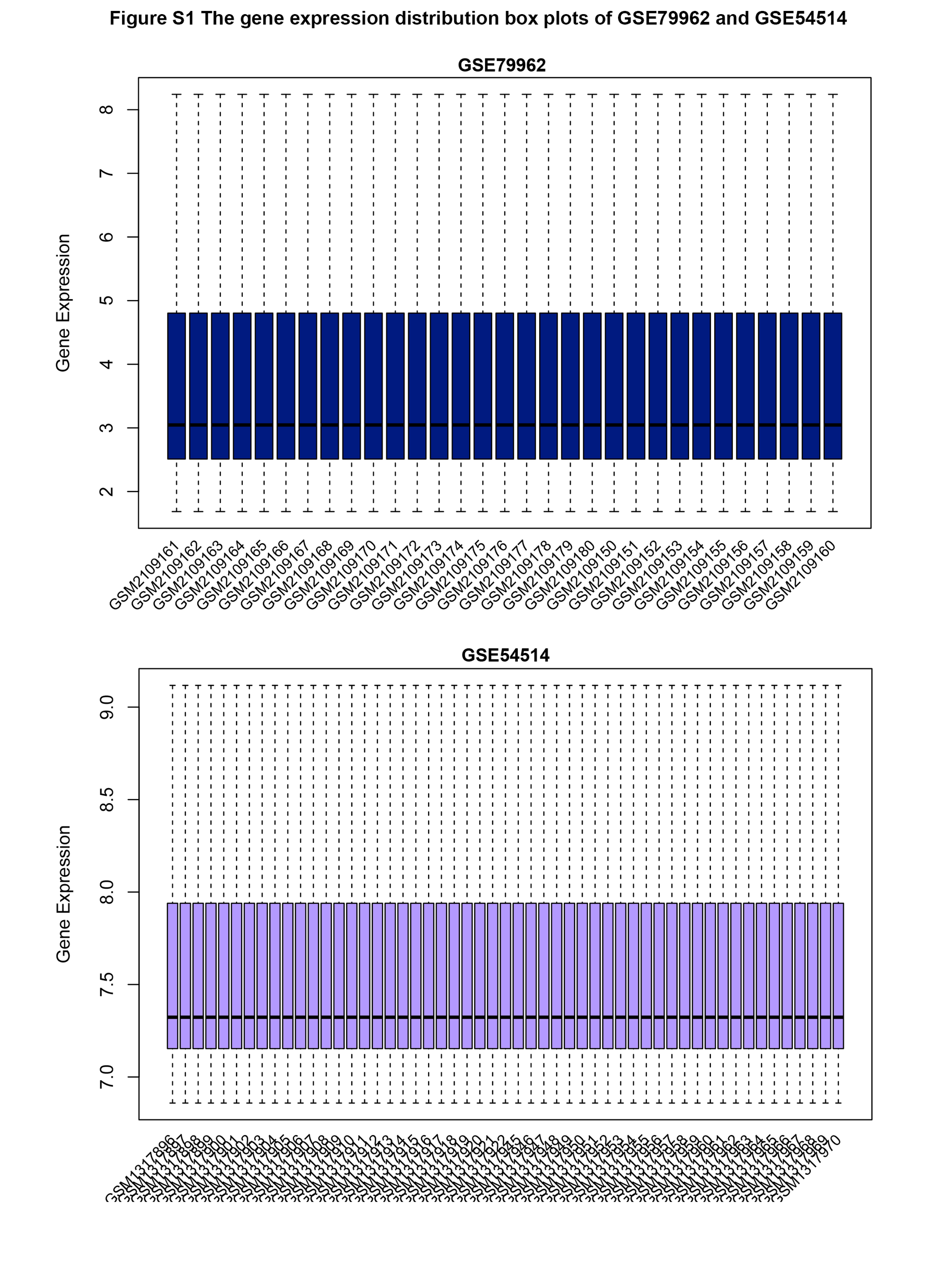

Supplement: Supplementary file 1 [file Image_1.TIF]

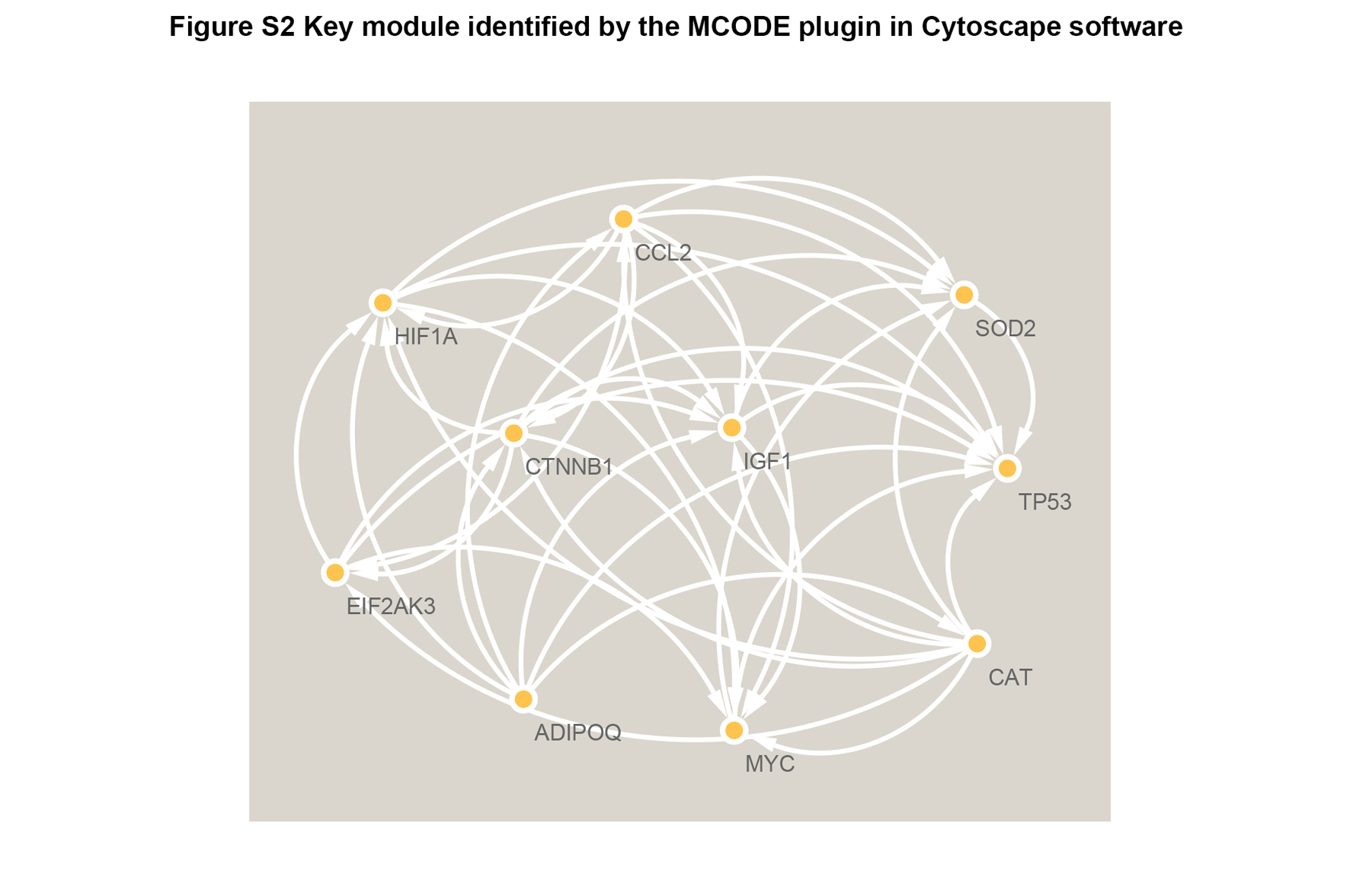

Supplement: Supplementary file 2 [file Image_2.TIF]

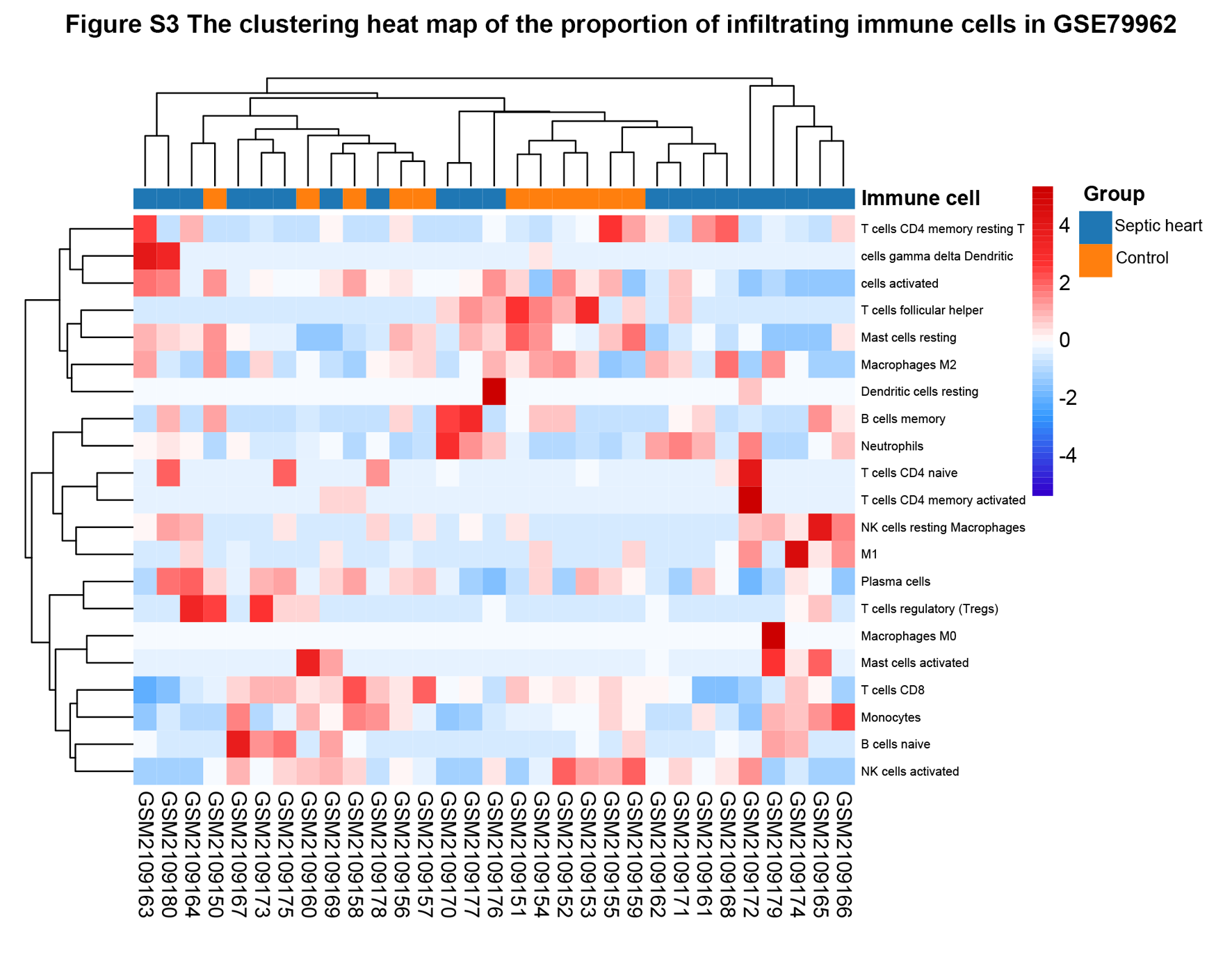

Supplement: Supplementary file 3 [file Image_3.TIF]
